# On-Farm Mitigation of Transmission of Tuberculosis from White-Tailed Deer to Cattle: Literature Review and Recommendations

**DOI:** 10.1155/2012/616318

**Published:** 2012-09-06

**Authors:** W. David Walter, Charles W. Anderson, Rick Smith, Mike Vanderklok, James J. Averill, Kurt C. VerCauteren

**Affiliations:** ^1^National Wildlife Research Center, Animal and Plant Health Inspection Services, United States Department of Agriculture, 4101 LaPorte Ave, Fort Collins, CO 80521, USA; ^2^US Geological Survey, Pennsylvania Cooperative Fish and Wildlife Research Unit, Pennsylvania State University, 403 Forest Resources Building, University Park, PA 16802, USA; ^3^Missouri Department of Conservation, Resource Science Division, 551 Joe Jones Boulevard, West Plains, MO 65775, USA; ^4^Animal Industry Division, Michigan Department of Agriculture and Rural Development, Lansing, MI 48909, USA

## Abstract

The Animal Industry Division of the Michigan Department of Agriculture and Rural Development (MDARD) has been challenged with assisting farmers with modifying farm practices to reduce potential for exposure to *Mycobacterium bovis* from wildlife to cattle. The MDARD recommendations for on-farm risk mitigation practices were developed from experiences in the US, UK and Ireland and a review of the scientific literature. The objectives of our study were to review the present state of knowledge on *M. bovis* excretion, transmission, and survival in the environment and the interactions of wildlife and cattle with the intention of determining if the current recommendations by MDARD on farm practices are adequate and to identify additional changes to farm practices that may help to mitigate the risk of transmission. This review will provide agencies with a comprehensive summary of the scientific literature on mitigation of disease transmission between wildlife and cattle and to identify lacunae in published research.

## 1. Introduction

Bovine tuberculosis (TB) is an infectious disease caused by the bacterium, *Mycobacterium bovis*, that affects both domestic and wild animals worldwide. In Michigan, TB was discovered in free-ranging white-tailed deer (*Odocoileus virginianus*) following the 1994 firearm deer season [1] in portions of Alpena, Alcona, Oscoda, and Montmorency counties, now referred to as Michigan's TB core area. Between 1994 and 2010, 50 cattle (*Bos taurus*) farms have tested positive for TB in 7 counties, with 40 of the farms being beef and 10 being dairy. Although considerable deer reductions have occurred within Michigan's TB core area since 1994, new and recurring infections continue to plague Michigan farmers.

Wildlife reservoir hosts for *M. bovis*, other than white-tailed deer, include the brushtail possum (*Trichosurus vulpecula*) in New Zealand and European badger (*Meles meles*) in the United Kingdom [2–4]. Other wildlife that have been found infected with *M. bovis* in Michigan include elk (*Cervus elaphus*), coyote (*Canis latrans*), raccoon (*Procyon lotor*), black bear (*Ursus americanus*), bobcat (*Lynx rufus*), Virginia opossum (*Didelphis virginiana*), red fox (*Vulpes vulpes*), and domestic cat (*Felis catus*; [5, 6]). Research examining transmission of *M. bovis* in Michigan has focused on deer-cattle interactions because of high deer densities and feeding practices that lead to deer congregating in high densities at feeding sites [7, 8]. It is believed that if *M. bovis* were eliminated from deer then it would not be sustained in other wildlife species [9] and could be eradicated from cattle.

 The Animal Industry Division of the Michigan Department of Agriculture and Rural Development (MDARD) created a Wildlife Risk Mitigation Action Project (WRMAP) to assist farmers with modifying farm practices to reduce potential for exposure to *M. bovis* from wildlife to cattle. The WRMAP recommendations are based on on-farm wildlife risk mitigation practices used elsewhere in the US [9, 10] and those in the UK [11, 12]. Several farm practices that have been identified increase the risk of transmission of *M. bovis* such as livestock feed (e.g., hay, grain, and silage) storage, livestock access to daytime cover used by wildlife, and cattle access to standing water sources (e.g., swamps, ponds) commonly used by wildlife [13] and additional factors have been identified in the current review. For a detailed review of past and current *M. bovis* eradication efforts in Michigan and surveillance of white-tailed deer see Okafor et al. [14]. 

The objectives of this paper were to review the present state of knowledge of *M. bovis* excretion, transmission, and survival in the environment and the interactions of wildlife and cattle, with the intention of determining if the current recommendations by MDARD on farm practices are adequate and to identify additional changes to farm practices that may help to mitigate the risk of transmission. We used numerous databases (e.g., WorldCat, JSTOR, DigiTop, Web of Knowledge) in our review of published literature on survival of *M. bovis* in the environment and surveillance for *M. bovis* in mammals. Additionally, we examined gray literature (e.g., state, federal reports) to further determine any additional factors that may contribute to WRMAP recommendations. 

## 2. Results

### 2.1. Routes of Transmission of *M. bovis*

The possible routes of infection of *M. bovis* are related to size, structure, and viability of bacilli in the reservoir and subsequent transmission pathways. *Mycobacterium bovis* bacilli are 1–4 *μ*m long, small, and nonmotile rods that are characterized by a waxy mycolic acid cell wall that offers protection and the ability to survive outside of the host [15]. Routes of transmission of *M. bovis* are likely dependent upon biology of the host animal as well as environmental variables for areas the host occupies. The high prevalency of pulmonary infections in Eurasian badger and laboratory studies strongly supports inhalation of infectious aerosol particles as the principal infection route [16–18]. Bite wounds were also a source of infection of Eurasian badgers with *M. bovis* and more common in males than females as males are territorial often leading to aggressive defense of territories [19, 20]. The culture of *M. bovis* from nine of 25 tracheal washings of brushtail possum would suggest a respiratory route of infection perhaps exacerbated by self-grooming and pouched young that led to a constant source of infection (i.e., discharging fistulae on an individual possum; [21]). The most commonly affected tissues of white-tailed deer infected with 5 × 10^3^ cfu (colony-forming units) *M. bovis* would suggest an oral route of transmission through the sharing of feed [22]. 

 Although many routes of transmission of *M. bovis* between and among animal species have been investigated, the minimum infective dose of *M. bovis* has been difficult to quantify. Ingestion of as few as 5 × 10^3^ cfu of *M. bovis* cattle calves resulted in establishment of infections and lesions [23], whereas 1 × 10^7^ and 1.3 × 10^6^ cfu of *M. bovis* administered orally was needed to establish infections in cattle and sheep, respectively in other studies (Sigurdson 1945, Francis 1947 as cited in [23]). The minimum infective dose for Eurasian badger was found to be <10 cfu of *M. bovis* through the endobronchial route for latent infection and 10^3-4^ cfu for generalized infection [24, 25]. A dose as small as 20 cfu of *M. bovis* administered intratracheally to 10^7^ cfu of *M. bovis* injected intramuscularly was found to cause severe tuberculosis in brushtail possum [26, 27]. A minimum infective dose of *M. bovis* necessary to cause TB and the dose actually encountered by wild animals and cattle is difficult to determine definitively, however, most likely routes of transmission of *M. bovis* have been determined for major reservoirs of TB.

### 2.2. Modes of Transmission of M. bovis

#### 2.2.1. Transmission of *M. bovis* between Captive and Wild Cervids 

Ten captive cervid herds have tested positive for *M. bovis *isolates since captive cervid regulations were implemented in 1999 that included 4, 1, 1, 1, and 3 herds in Michigan, Kansas, New York, Nebraska, and Indiana, respectively [14, 28]. After several elk were identified as infected with *M. bovis* on a game ranch in Montana, infection was found in adjacent herds of free-ranging mule deer (*O. hemionus*) with transmission presumed to have occurred by direct contact along the adjoining fence-line [29]. Surveillance from November 1993 to January 1994 found that 2 of 41 free ranging mule deer infected with *M. bovis* likely from direct fence-line contact with positive elk [29]. Evidence of fence-line contact between free-ranging deer and captive deer was documented in Michigan although at a low occurrence [30]. Transmission of TB between wild and captive cervids seems unlikely due to the low occurrence of documented contacts studied at captive facilities.

#### 2.2.2. Transmission of *M. bovis* between Captive Cervids and Cattle 

 There has been no direct observation of *M. bovis* transmission between captive cervids and cattle although direct contact between the species has been reported in surveys of farmers but the research findings are less supportive. In a US Department of Agriculture (USDA) survey of farmers, the percentage of dairy operations in the U.S. that reported physical contact between cattle and cervids (either captive or free-ranging) ranged from 20.9% in the west to 51.6% in the east, with 90.8% of survey respondents reporting that cattle “could possibly or sometimes have face-to-face contact with deer” [28]. In that survey, 72.6% reported contact with free-ranging cervids but only 3.2% of beef cow-calf operations reported fence-line interactions or commingling with captive cervids [28]. As nationwide occurrence of captive cervids infected with *M. bovis* is very low, transmission of *M. bovis* between captive cervids and free-ranging wildlife or cattle may be of little importance. The ability of farmers or ranchers to control contact between cattle and captive cervids (i.e., double fence around captive cervids) would likely prevent transmission of TB. 

#### 2.2.3. Interactions between Wildlife and Cattle 

Initially, *M. bovis* transmission from wildlife to cattle (direct) was considered the major route of infection on cattle farms in Michigan [9]. Hill [31] observed only one direct interaction between deer and cattle that involved a female and 2 fawns running by a single cattle and coming within 5 m. Although observations on direct contact was not an objective of the study, elk use of farms around Riding MB National Park in MB, Canada, was positively associated with distance to protected area (i.e., parks) and forested areas that provided suitable cover for elk [32]. A study on cervid damage in Manitoba, Canada identified a reduction in elk damage to stored feed of nearly 50% after farms fenced stored feed but 18% of farmers still observed white-tailed deer inside the barrier fencing [33]. The majority of observations or surveys were of farmers that have had their farms test positive for *M. bovis* suggesting that an environment-to-cattle (indirect) transmission of *M. bovis *from wildlife should be the focus of efforts to mitigate transmission of *M. bovis* from deer to cattle and the focus of research on *M. bovis* in the future. Contrary to initial beliefs that direct contact was responsible, indirect contact may be just as important of a route of transmission of *M. bovis* on farms, however, information on shedding of *M. bovis* by wildlife should be considered. 

#### 2.2.4. Shedding of *M. bovis* from Infected Animals 

Survival of *M. bovis* in the environment varies from days to months depending on environmental conditions, but there is scant information on routes of shedding or the concentration of *M. bovis* in excretions from infected wildlife. Hence, the probability of direct and indirect transmission to cattle is difficult to assess. Details on shedding of *M. bovis* from naturally infected animals of the taxonomic groups commonly found on farms would assist in understanding the probability of transmission (Table 1). In the subsequent section, only reports where “shedding” was defined as the culture *M. bovis* from saliva, feces, urine, or exudates are reviewed. Shedding that was presumed or otherwise not substantiated was not reported as shedding in subsequent sections. 

(1) *Shedding by Avian Species.* Shedding of *M. bovis* in avian species has only been investigated in a few species with low to minimal amounts of shedding reported (Table 1). Fecal samples were negative for *M. bovis* in laboratory experiments with American crow (*Corvus brachyrhynchos*), European starling (*Sturnus vulgaris*), and mallard ducks (*Anas platyrhynchos*; [34, 35]). Pigeon (*Columba livia*) had *M. bovis* present in excrement for at least 60 days after inoculation [36]. With the wide range of avian species and their presence on farms, additional research on shedding of *M. bovis* appears to be warranted.

(2) *Shedding by Marsupialia.* Shedding of *M. bovis* has been reported for brushtail possum in New Zealand and Virginia opossum in North America (Table 1). Diegel et al. [37] inoculated 8 opossum (4 orally, 4 intramuscularly) in the laboratory, 5 were positive for *M. bovis* one month after-inoculation, and shedding of *M. bovis* was observed. Oral infection of 4 Virginia opossum resulted in feces positive for *M. bovis* 1 day and 30 days after-inoculation but these occurred in different individuals [37]. Laboratory aerosol inoculation of 12 Virginia opossum (6 high dose, 6 low-dose) resulted in low shedding in one individual in the low dose group on day 60 after -inoculation [38]. Six opossum orally inoculated with high and low doses of *M. bovis* had “uncommon” fecal shedding at both doses suggesting that transmission of *M. bovis* by fecal shedding or direct contact was not likely [38]. Palmer et al. [39] documented excretion of *M. bovis* in saliva or nasal secretions in raccoon but not in urine or feces leading the authors to claim raccoon were not likely reservoirs of *M. bovis*. Diegel et al. [37] believe more research is needed on opossum as reservoirs.

(3) *Shedding by Insectivora/Rodentia.* Only one study has investigated shedding of *M. bovis* by small mammals (Table 1). Clarke et al. [40] inoculated meadow voles (*Microtus pennsylvanicus*) with *M. bovis* in doses of 5 × 10^3^ cfu orally (OR group; *n* = 12) or 1 × 10^5^ cfu intranasally (IN groups; *n* = 12) and positive fecal cultures were detected on day 1 in 9 and 8 meadow voles, respectively. Of the surviving meadow voles at day 30, none of 5 in the OR group and 2 of 5 in the IN group had positive fecal cultures. The ability of small mammals to act as reservoirs is not currently supported by field data but does require further research [40]. Clarke et al. [40] inoculated house mice (*Mus musculus*) and Norway rats (*Rattus norvegicus*) with *M. bovis* but fecal shedding was not detected.

(4) *Shedding by Carnivora.* A plethora of research has been conducted on shedding of *M. bovis* in carnivores (Table 1) following the demonstration that European badgers were the primary reservoir in the UK and Ireland. In the laboratory, intravenous inoculation of European badgers with *M. bovis* resulted in shedding of the bacteria in urine and feces for as long as 1,305 days [41]. Samples from free-ranging European badgers were positive for *M. bovis* in urine, feces, sputum, and bite wounds [42]. The proportion of naturally infected badgers excreting *M. bovis* in urine and feces is low, however, with 11.4% and 3.8%, respectively, and the concentration of bacilli in the excreta was also very low [25]. Lugton et al. [43] found *M. bovis* present in feces or colonic swabs from 10 of 63-sampled free-ranging feral ferret (*Mustela furo*). In the laboratory, various doses of *M. bovis* inoculation in raccoon resulted in shedding in saliva and nasal secretions but not in urine or feces [39]. Preliminary results found that prevalence of *M. bovis* was 3% in raccoon on farms depopulated of cattle following detection of *M. bovis* from 2006 to 2008 in Michigan [44]. No shedding of *M. bovis* was found in oral, nasal, or fecal samples from free-ranging coyote in Michigan [45]. Further research on shedding of *M. bovis* in free-ranging carnivore species seems warranted to further understand their role in TB in Michigan.

(5) *Shedding by Artiodactyla.* Shedding of *M. bovis* has been studied extensively in a variety of hoofed mammals as a result of recurring infections in cattle and the reservoir host in the US white-tailed deer (Table 1). Low or no shedding of *M. bovis* was documented in free-ranging red deer in New Zealand and marsh deer (*Blastocerus dichotomus*) in Brazil, respectively [46, 47]. Laboratory inoculations of domestic cattle and white-tailed deer demonstrated that *M. bovis* can be shed in saliva, nasal secretions, urine, and feces [48–50]. White-tailed deer in the low-dose group (2 × 10^3^ cfu; *n* = 4) and high dose group (2 × 10^5^ cfu; *n* = 4) had tonsillar swabs positive for *M. bovis* in one (at day 14) and 2 (at day 35) deer, respectively. Oral swabs for white-tailed deer yielded *M. bovis* at 63 and 80 days for one deer in the low dose group from no deer in the high-dose group [48]. Of 4 white-tailed deer that received 2 × 10^8^ cfu of *M. bovis* in by intratonsillar instillation, detection of *M. bovis* in tonsillar, oral, nasal, and rectal swabs was possible in at least one deer at 63, 90, and 113 days after-inoculation [49]. Calves infected intranasally or intratracheally with the low dose (1.1 × 10^4^ cfu–2.0 × 10^4^ cfu; *n* = 6 per route) and high dose (1.7 × 10^6^ cfu–4.0 × 10^5^ cfu; 6 per route) of *M. bovis* resulted in more severe pathology and more episodes of shedding in the high dose calves 80 days post-inoculation [50]. Free-ranging cervids have long been implicated in transmission of *M. bovis* and shedding has substantiated their ability to maintain TB and infect cattle when overlap of range has been documented.

### 2.3. Survival of *M. bovis* under Experimental Conditions 

 Since the 1930s, a variety of investigations have been published on what may influence survival of *M. bovis* (referred to *Bacilli tuberculosis* in earlier papers), including examination of artificial and natural inoculations and variation in temperature, moisture, and substrates. Numerous factors contribute to the survival or loss of viability of *M. bovis* in the environment, and these have been investigated under both laboratory and natural environmental conditions. 

Williams and Hoy [51] examined persistence of *M. bovis* in Great Britain in artificially and naturally infected soil, cattle feces, and intestinal content. Guinea pigs were used to determine presence of *M. bovis* via inoculation with treatment material. Under natural shaded conditions on pastures, positive samples from cattle feces inoculated with *M. bovis* were detected up to 5 months post-application during winter but only up to 2 months during spring and summer. If the feces were shaded from sunlight, however, then positive samples were observed post-application for up to 4 months during summer and 6 months in autumn. All intestinal samples from tuberculous cattle spread on pasture were positive for *M. bovis* immediately after application but subsequently tested negative for up to 4 months. If stored in a cool cellar, artificially and naturally infected feces tested positive for 24 months and 12 months, respectively. Artificially infected liquid feces stored underground tested positive for at least 4 months.

#### 2.3.1. Survival of *M. bovis* in Soil-Related Substrates

Maddock [52] investigated the survival time of *M. bovis* in soil, cattle feces, and feces and soil mixtures exposed in open pastures of Great Britain. The viability of *M. bovis* on growing pasture grass was also explored. Porous pots were placed at ground level within an open pasture and filled with soil, feces, or a mixture of feces and soil; and *M. bovis *emulsion was subsequently added. Guinea pigs were used to determine presence of *M. bovis *via inoculation with treatment material. Soil, feces, and soil and feces-mixture treatments all tested positive for up to 6 months post-application. Sampling from the pots occurred from June to December. An emulsion of *M. bovis *material (tuberculous material collected from abattoirs) was sprayed on grass growing in a pasture at 3 different dilution levels (Plot I: 120,000 *M. bovis*/ft^2^; Plot II: 1.2 million; Plot III: 120 million). Plot III tested positive up to 49 days after application but plot I only tested positive for up to 14 days after application. The author noted (no measurements reported) there was heavy rain after application followed by heat and dryness during the grass experiment which was conducted from April to June.

Maddock [53] conducted field investigations from 1932 (grass and soil experiment) to 1933 (grass experiment only) as a continuation of the experiment published in Maddock [52] with the purpose of examining survival of *M. bovis *given annual variation in weather from the previous studies in 1932 and 1933. The average mean temperature for the months of July, August, and September, 1932 was 2.9°C higher than that of the same period in 1931. The maximum temperature in 1932 was 34.8°C, as compared with 24.4°C for 1931. Postapplication treatments tested positive for *M. bovis* up to 2, 5, and 5 months for soil, feces, and soil/feces mixture, respectively. The grass experiment was conducted from April to September in 1932 and from June to September 1933. During 1932, positives were detected at 14, 28, and 49 days after application for plots I, II, and III, respectively. The temperature (no measurements reported) during the summer of 1933 was noted as very hot and attempts were made to detect *M. bovis* on grass with application of dilutions of 5 million* B. tuberculosis*/ft^2^, 7 million, and 25 million. Only the plot inoculated with 7 million *B. tuberculosis*/ft^2^ had a positive detection, occurring at 63 days after application but not at 83 or 105 days after application.

Van Donsel and Larkin [54] investigated the survival of *M. bovis* in soil and on radishes and lettuce in raised garden plots (lined plywood boxes). Plots were outside under natural conditions during summer in Cincinnati, OH, USA. Soil and vegetables were sprayed or irrigated with either sewage effluent or sludge 2 weeks after planting with *M. bovis* cultures at 10^6^ cfu/mL. Culture media was used to determine presence of *M. bovis*. Effluent plots tested positive for *M. bovis* for up to 29, 13, and 35 days after application for soil, radishes, and lettuce, respectively. Sludge plots tested positive for *M. bovis* for 29, 10, and 35 days after application for soil, radishes, and lettuce, respectively. Contamination (e.g., mold) became too great in soil after 29 days to accurately identify *M. bovis* in samples.

Duffield and Young [55] investigated survival of *M. bovis* in artificially inoculated dry and moist soils and bovine feces under various environmental conditions for 32 weeks in tropical northern Australia. Environmental conditions included laboratory (mean daytime temperature = 23°C) and sun (mean daytime temperature = 43°C), shade (80% shade cloth, mean daytime temperature = 34°C), and dark (containers covered with aluminum foil, mean daytime temperature = 32°C) in the external environment. Identification of *M. bovis* was performed using culture media, drug sensitivity, and guinea-pig (*Cavia porcellus*) inoculation [55]. All tested environmental conditions were conducted using sterile and non-sterile substrates. Substrates were dry, sandy loam pH 6.3–6.7; sandy loam with 30% (v/w) moisture content; fresh feces from cattle. Substrates inoculated with 5 × 10^8^ cfu of an *M. bovis* field strain were used to inoculate 50 g of substrate. Samples positive for *M. bovis* were identified up to 4 weeks after inoculation for nonsterile substrates in dry and moist soils in shade, dark, and laboratory conditions. Samples positive for *M. bovis* were also observed up to 4 weeks after inoculation in sterile-moist soil in shade and dark conditions. Samples positive for *M. bovis* were not observed in the remaining combinations of tested substrate and environmental conditions. The authors concluded that the low survival of *M. bovis*, as compared to other studies [51, 52], was due in part to higher temperatures during the study.

Jackson et al. [21] investigated the survival of *M. bovis* in New Zealand using inoculated cotton ribbons placed in brushtail possum dens, open pasture, and the forest floor. The inoculum was 50 *μ*L of *M. bovis *at a concentration of 1–5 × 10^8^ organisms per milliliter. Samples positive for *M. bovis* were determined using culture media. Samples positive for *M. bovis* were observed for up to 4 days on open pasture during spring and summer, and on the forest floor during summer. Samples positive for *M. bovis* were observed for up to 7 days underground in dens during summer and 14–28 days on forest floor during winter. Authors concluded that bacteria survival increases as temperature decreases and has higher survival during spring and winter than summer.

Young et al. [56] investigated the survival of *M. bovis *Bacillus Calmette-Guérin (BCG) in soil collected from farms that tested positive for *M. bovis* in Ireland. Soil samples were monitored in a laboratory after inoculation with 10^8^ cells of* M. bovis *BCG. Soil samples were stored at 15, 20, 25, and 37°C with various water moisture levels of −1,600, −400, −66, −33, −20, and −10 kPa matric potential. Samples positive for live organisms of *M. bovis* were detected using culture media and Polymerase Chain Reaction (PCR). Experiments revealed that *M. bovis* BCG survival was optimal at 37°C with moist soil (−20 kPa; 30% [vol/wt]). The authors concluded that *M. bovis *can persist in the farm environment for up to 10 days outside of its hosts and survival was influenced by climatic factors. 

#### 2.3.2. Survival of *M. bovis* in Feces

Recovery of *M. bovis* from cattle feces that were artificially infected with (emulsified tuberculous bovine lung lesions) and spread on pasture was recovered on pasture 2–4 months later with survival longer during winter (5 months) than during summer (<2 months), although larger doses of *M. bovis *inoculums were used for winter than summer experiments [51]. *M. bovis* was recovered for naturally infected feces stored in cool dark conditions after 12 months and for 2 years in experimentally infected feces stored in a similar manner [51]. 

Tanner and Michel [57] examined the survival of *M. bovis* in South Africa using naturally infected lung or lymph node tissue and artificially inoculated cattle feces with 8 × 10^7^ organisms. Fecal samples were placed on polystyrene trays and placed in shaded or nonshaded cages (protected from ants and animals) in dry and moist soil areas and trees across 4 seasons (winter, spring, summer, and autumn). A set of fecal samples were also placed 20 cm underground in moist soil plots. Positive samples of *M. bovis* were identified via culture media and PCR. No positives were observed beyond 5 days for buried samples. For naturally infected samples in nonshaded conditions during winter, *M. bovis* was detected for as long as 27 days in moist soil areas but as long as 42 days in the shade. During summer, *M. bovis* was detected for as long as 14 days in shaded moist soil areas but only up to 5 days in trees and dry soil plots. During spring, *M. bovis* was detected for as long as 14 days in moist soil areas but only up to 5 days in shade and dry soil conditions. During autumn, *M. bovis* was detected for as long as 7 days in moist soil plots but only 2–5 days in other conditions. 

Tanner and Michel [57] also investigated artificially infected samples from cattle feces in non-shaded conditions. During winter, *M. bovis* was detected for as long as 27 days in moist and dry soil plots but only for up to 5 days in shade under trees. During spring, *M. bovis* was detected for as long as 21 days in moist soil areas but only up to 2 days in trees. During summer, *M. bovis* was detected for 5 days in shade under trees, dry soil, and moist soil plots. During autumn, *M. bovis* was detected for as long as 14 days in moist plots but only 5–7 days in other conditions. For artificially infected fecal samples in shaded conditions during winter, *M. bovis* was detected for as long as 14 days in moist and shade tree plots but only up to 5 days in dry soil conditions [57]. During summer, *M. bovis* was detected for only up to 5 days in shade, dry soil, and moist soil plots. During spring, *M. bovis* was detected for as long as 21 days in moist and shade tree plots but only up to 14 days in dry soil. During autumn, *M. bovis* was detected for as long as 14 days in dry and moist plots but only up to 7 days in shade tree plots. The authors concluded that moisture and temperature greatly affected the duration of survival of *M. bovis *in the environment.

#### 2.3.3. Survival of *M. bovis* in Feed

Palmer and Whipple [58] examined the survival of *M. bovis* on feedstuffs often used for feeding white-tailed deer in Michigan such as apples, corn, potatoes, and sugar beets. Each sample was inoculated with 1 mL volume of a bacterial suspension containing 1.1 × 10^6^ cfu of *M. bovis* then randomly placed in storage with 1 of 3 environmental temperatures (−20°C, freezing; 8°C, refrigeration; 23°C, room temperature). Samples positive for *M. bovis* were identified using culture media and genetic isolation. Survival of *M. bovis* was for at least 7 days on all feedstuffs at all temperatures tested. At the termination of the study (112 days), *M. bovis* could be recovered from all feedstuffs at −20°C, from apples, corn, hay, and potatoes at 8°C, and from apples, corn, and potatoes at 23°C. Carrots and sugar beets were the least favorable substrate for survival, however, *M. bovis* could be recovered from both at 112 days at −20°C and 14 and 84 days, respectively, at 8°C. 

 Fine et al. [59] investigated the survival of 5 × 10^4^ cfu *M. bovis* artificially applied to corn, hay, soil, and water exposed to natural conditions within an outside exclosure in Michigan. Substrates were inoculated with 50,000 cfu of *M. bovis* extracted from bovine lymph node that tested positive for *M. bovis*. Samples positive for *M. bovis* were identified via culture media and genetic isolation. During the autumn/winter sampling period, samples positive for *M. bovis* were identified up to 37, 43, and 28 days after inoculation in corn, hay, and soil substrates, respectively. During the winter/spring, samples positive for *M. bovis* were observed up to 26, 26, and 88 days after inoculation for corn, hay, and soil substrates, respectively. During the spring/summer, samples positive for *M. bovis* were observed up to 3, 0, and 11 days post inoculation in corn, hay, and soil substrates, respectively. There was no significant substrate affect, but *M. bovis* had a greater persistence in water in summer. Shade increased persistence of *M. bovis*, except during winter/spring. The authors concluded that survival of *M. bovis* decreased as temperatures increased, solar radiation intensified, and evapotranspiration increased. 

#### 2.3.4. Survival of *M. bovis* in Water

Survival of *M. bovis* in water has been documented at up to 400 days in running water even though dilutions in running water will decrease the overall numbers of the bacteria in samples collected [60]. Fine et al. [59] documented survival of *M. bovis* in artificially contaminated water that was exposed to natural environmental conditions and showed that the bacilli could survive for 58, 21, and 48 days during autumn/winter, winter/spring, and spring/summer, respectively. The isolation of *M. bovis* from water troughs or natural water sources exposed to naturally infected animals has not been reported. Michel et al. [61] housed experimentally infected African buffalo (*Syncerus caffer*) with naïve buffalo that shared water troughs but were unable to find *M. bovis* in water samples even though buffalo tested positive several months post-trial. Transmission of *M. bovis* in water either by infected cattle or wildlife is unlikely based on infective dose needed and conditions necessary for survival of an infective dose in water.

#### 2.3.5. Survival of *M. bovis* in relation to Farm Practices

 Studies examining the survival of *M. bovis* in the natural environment rarely have attempted to determine the half-life of *M. bovis* survival, however, they are very informative and hold the key to some of the variables that determine viability in the environment. High temperatures lead to decreased survival of *M. bovis* [21, 57, 59], and vice versa, lower temperature lead to higher probability of survival [21, 62]. Moisture content seems correlated with survival such that lower available moisture leads to cell death [56, 57, 59]. Under the conditions investigated, environmental survival of *M. bovis* ranges from only a few days to many months with survival influenced by exposure to sunlight, temperature, soil type, moisture content, pH, and whether the bacilli were in soil, water, feed stuffs, or air. The intricate combination of factors involved in survival of *M. bovis* on farms makes knowledge of these intricate relationships integral to understanding transmission of TB in regions that have wildlife and domestic animal host.

Recurring cases in Ireland were related to no mineral lick supplements being supplied to cattle (i.e., nutritional effects), purchase of a bull (i.e., cattle movements among farms), presence of cubicle housing, rough grazing areas (i.e., poor quality soil), and presence of European badgers on the farm [63]. Buying in cows (i.e., purchasing cattle from an outside source as opposed to operating a closed herd), purchase of >50 cattle during the study period, and storing manure for at least 6 months increased chance of transient (i.e., initial *M. bovis* exposure) breakdown on farms; high stocking density (≥3 cattle/ha) decreased odds of *M. bovis* although stocking density was a categorical variable (i.e., low, intermediate, high) based on the 5-year average stocking density relative to the area of grassland on the farm [64]. Buying in cows and the use of concrete storage bins covered by plastic sheets (hereafter referred to as silage clamps) on the farm increased the risk of repeated *M. bovis* infection; farming of a mixed herd (i.e., both dairy and beef cattle) and providing hay to cattle decreased chance of repeated *M. bovis* infection [64]. 

(1) *Livestock Grazing.* Grazing cattle tend to ingest soil when grazing on vegetation that is short suggesting that overstocking or overgrazing of areas can increase transmission of *M. bovis* [3, 65]. Within-herd transmission has been suggested to be greater with cattle housed indoors rather than in pastures [65]. Poorly ventilated cattle housing, high density of cattle, high humidity, and low sunlight is an ideal environment for survival of *M. bovis* and lead to increased potential for transmission [65, 66]. Farm yard (composted) manure must be exposed to a mean temperature of 60–70°C for 3 weeks during composting to destroy *M. bovis* bacilli, but most solid dung heaps were not able to reach that high temperature (Hahesy 1996 in [65]). Survival in slurry can vary from 10 weeks to 6 months with storage temperature being the key factor determining survival (Hahesy et al. 1992 in [65]). Survival in slurry can last 17 months at 40–45°C (Turgenbaev 1986 in [65]) but only 30 days at 54°C (Vera et al. 1988 in [65]).

(2) *Feeding of Livestock.* Several *Mycobacterium* spp. have been documented to lay dormant for prolonged periods under anaerobic conditions prior to reactivation of endogenous infection [67–69]. In such conditions, *M. bovis* develops a thick cell wall and enters a nonreplicating or dormant state [65, 67]. The process of silage production (i.e., zero oxygen concentration within days of ensiling) may induce *M. bovis* to enter a dormant state [65, 67]. As the temperature during ensiling and grass storage can reach 30°C [70], and the optimal temperature for *M. bovis* survival is 37°C [71], silage or grass clamp preparation and storage provide ideal conditions for *M. bovis* survival and should be investigated as a possible source for *M. bovis* infections on farms in Michigan. 

(3) *Stored Feed.* Feed storage and fencing to exclude wildlife have been considered the primary method to decrease the transmission of *M. bovis* on farms [59, 72]. From 2000 to 2004, 8 of 12 farms surveyed stored round hay bales outside and were unprotected from wildlife [73]. Stored feed and supplemental feed has been implicated in contributing to transmission of *M. bovis* in white-tailed deer in Michigan [8]. Results from modeling efforts indicated that prior to the 1998 baiting ban in Michigan, prevalence of *M. bovis* on farms had a positive relationship with the percentage of sites that provided fruits and vegetables (i.e., apples, carrots, sugar beets) but a negative relationship with percentage of sites that provided grain [8]. Indirect transmission by contaminated feed was documented in a laboratory setting by feeding leftover grain consumed by deer infected with *M. bovis* to naïve deer for 120 days resulting in infection of all naïve deer (*n* = 4; [22]). Palmer and Whipple [58] documented that *M. bovis *was present on various supplemental feeds at 3 temperatures (−20°C, 8°C, 23°C) for at least 7 days and at 23°C *M. bovis* was isolated for as long as 112 days. Silage appeared to be more accessible to European badgers in Ireland and 100% of the farms that experienced persistent breakdowns of *M. bovis* fed grass silage [64].

(4) *Wildlife Activity on Farms.* The influence of wildlife activity on transmission of *M. bovis* depends on possible reservoirs or spillover hosts and their ability to transmit disease. Elk have been reported to use the same pastures, streams, ponds, cattle troughs, and mineral supplements as cattle near Riding Mountain National Park, Manitoba, Canada and were assumed to be responsible for the continued presence of *M. bovis* in the region [32]. Visits to farm yards and areas used by cattle were studied in Michigan where sixteen radiocollared white-tailed deer in the TB core area were monitored. Incursions into farm yards were infrequent and undertaken by few of the deer but 2 deer (12.5% of study subjects) were responsible for 80% of the visits to farm yards [74]. Several medium-sized mammals (i.e., European badgers, brushtail possum, raccoon) have been observed using farms, feeding troughs, and pastures. European badger feces were observed in feed troughs on cattle farms in the UK and shedding in feces has been frequently reported [11, 42]. Brushtail possum avoided direct contact with cattle but used pastures occupied by cattle and the access of cattle to areas where possum denned was directly associated with transmission of *M. bovis* from brushtail possum to cattle [75]. Hill [31] documented 273, 248, and 112 indirect interactions with cattle by white-tailed deer, other mammals (raccoon, rabbit, coyote, and Virginia opossum), and eastern wild turkey (*Meleagris gallopavo*), respectively, over 2 years in Michigan. Indirect interactions between cattle and white-tailed were dominated by deer using pastures and silage storage areas; deer fed from hay racks or silage troughs on only one occasion [31]. Most interactions by mammals other than deer were observations passing through pastures without visiting cattle feeding areas.

(5) *General Farm Practices.* Mixed dairy and beef enterprises were at greater risk than nonmixed operations for contracting *M. bovis* in Italy likely due to the cattle movement among farms which was considered a major risk factor [76]. In the UK, Reilly and Courtenay [64] found increased odds of both transient breakdown (i.e., cattle on a farm only positive once for *M. bovis*) and persistent breakdown (i.e., cattle on a farm repeatedly positive for *M. bovis*) on farms that imported cows but only transient breakdowns for farms that purchased >50 head of cattle. Several studies have indicated that movement of cattle between farms was a contributing factor to *M. bovis* transmission in Europe [63, 77]. In England, increased risk of TB was associated with movement of cattle onto farms from markets or farm sales suggesting an outside source of TB infection [77]. Importing >50% of cattle annually, however, did not increase odds of a farm coming down with *M. bovis* in Michigan [13]. In North America, a survey in 17 of the Nation's major dairy states found 4 out of 10 dairy operations surveyed between 1996 and 2006 brought cattle onto the operation to replace culled cows or to increase herd size [28]. For large dairy operations of ≥500 head, percent of new additions ranged from 1% for preweaned calves to 61.6% for any beef or dairy cattle. For small dairy operations of <100 head, percent of new additions ranged from 1% for beef heifers and cows to 35.6% for any beef or dairy cattle. Fine [73] surveyed 13 Michigan farmers (4 dairy, 7 beef, 2 small beef feeder) with mean herd size of 74 cattle (range: 14–239) that had cattle test positive for *M. bovis* between 2000 and 2004. “About half” of the farms surveyed purchased 100% of their cattle from outside sources suggesting that cattle importation increases likelihood of cattle testing positive for *M. bovis* that is similar to finding in other countries. 

### 2.4. Destruction of *M. bovis*

Although the previous sections detailed survival and hence, death of *M. bovis* under various conditions (i.e., temperature, humidity, and ultraviolet light), killing or active destruction of bacilli responsible for TB would be beneficial in farm buildings or structures that previously housed cattle positive for *M. bovis*. Although decontamination of surfaces is common for human practices [78], we found only one study that directly investigated the destruction of *M. bovis* with volatile chemicals [79]. Scanlon and Quinn [79] evaluated the ability of 5 volatile chemicals to inactivate the bacteria in cattle slurry. Acetone at concentrations of 22.5% inactivated *M. bovis* in under 24 hours and a 1.0% concentration of ammonium hydroxide inactivated *M. bovis* after 36 hours. Chloroform, ethyl alcohol, and xylene at concentrations of 0.5%, 17.5%, and 3%, respectively, inactivated *M. bovis *within 48 hours [79]. Due to the cost, safety considerations, and risk of environmental pollution from volatile chemicals, use of chemicals to inactivate *M. bovis* should be critically evaluated and is likely not a practical means of treating farms previously found to be positive for *M. bovis* [79]. 

## 3. Discussion

### 3.1. Wildlife Risk Mitigation Project Strategy

The WRMAP was a collaborative effort between the Michigan Department of Agriculture and Rural Development, Michigan State University Extension, USDA (Veterinary Services, Wildlife Services), and the Natural Resources Conservation Service to help Michigan livestock producers protect their cattle from *M. bovis*. The WRMAP was intended to educate farmers on potential risky farm practices that may lead to *M. bovis* contamination and to identify measures to reduce their risk. After a farmer commits to a WRMAP, he/she implements the WRMAP followed by verification of the plan by technical experts. The WRMAP contains specific components that could reduce *M. bovis* transmission on farms that includes (1) livestock feeding practices, (2) feed storage, (3) livestock water sources, (4) wildlife activity, and (5) general farm practices.

Livestock grazing and feeding practices greatly influence transmission of disease because various factors that farmers use to efficiently feed cattle can contribute to survival of *M. bovis*. Grazing cattle can ingest soil when grazing on vegetation that is short as a result of overstocking or overgrazing of areas that can be addressed by farmers [3, 65]. Farmers often use manure or slurry as fertilizer for crops and these areas are then often used for cattle grazing. Manure and slurry must be exposed to the proper temperatures for long enough duration to destroy *M. bovis* bacilli prior to spreading on pasture or crop fields that are intended for grazing by cattle (Hahesy 1996 in [65]). Although crop fields and pastures are typically on open lands and in direct sunlight limiting survival of *M. bovis*, spreading improperly processed manure or slurry should be avoided. 

 The type of feed a farm supplies to cattle influences the amount of deer damage because deer preference for feed dictates their level of motivation to attain such feed [72]. Damage to feed could influence infection from deer and was considered an important mitigation measure in Minnesota programs. For example, herds surveyed in the Minnesota core TB zone had the most deer damage to silage (71%) followed by alfalfa (2nd/3rd cutting; 34%) and beet pulp (38%) with grass hay receiving the least amount of damage (2%; [72]). Feeding or storing of fruits and vegetables was associated with higher prevalence in deer because one deer may not consume an entire fruit or vegetable and if sufficiently contaminated has the potential to infect the next deer or cattle that consumed the partially consumed fruit or vegetable [8]. Furthermore, feeding grains was negatively associated with TB prevalence in deer [8] because it was easier to disperse thus decreasing the density of deer feeding in the same areas when compared to feeding hay. Farmers feeding bales of hay year-round would appear to be more at risk of contracting *M. bovis* because hay could be left uneaten, and if contaminated with *M. bovis*, they provide a source of disease transmission to other animals (i.e., cattle, deer).

Feed storage and fencing to exclude wildlife have been considered the primary method to decrease the transmission of *M. bovis* on farms in Michigan and Minnesota [14, 59, 72]. Increased efforts by the USDA and MDARD have provided hoop barns or deer-proof fencing to protect stored feed. Previous research has indicated that protection of feed should be a primary focus of the WRMAP because indirect transmission of *M. bovis* can occur from up to 112 days under the proper environmental conditions. Although less likely, an additional practice not included in WRMAP includes potential for importing stored silage or hay and the likelihood of introducing *M. bovis* from outside sources of feed. Previous studies documented that stored manure and feed increased the risk of *M. bovis* infection [64] but were unable to document if feed purchased from outside sources could be a route of transmission. 

Although *M. bovis* has been shown to survive in water for extended periods, the role of transmission of *M. bovis *via water sources remains unknown [80]. Current risk mitigation measures require farmers to exclude cattle from ponds or water bodies that may be accessible to wildlife and exclude wildlife from cattle water troughs or water storage tanks [14, 81]. Although *M. bovis* has been shown to survive in water for extended periods (i.e., days to months) under artificial conditions and is currently within the WRMAP, the role of transmission of *M. bovis *via water sources remains unknown and likely highly dependent upon the proper combination of climatic conditions, water properties (i.e., standing or running water), level of contamination, infective dose, and dilution.

Wildlife activity has been linked to infections from wildlife to cattle for recurring cases of *M. bovis* on cattle farms in Michigan, USA, and the UK but the exact route is difficult to determine [14, 82, 83]. The influence of wildlife activity on transmission of *M. bovis* has been the focus of mitigation measures to limit access to farms for badger in the UK and Ireland [11, 63], brushtail possum in New Zealand [75], white-tailed deer in Michigan and Minnesota, USA [14, 81], and elk in Canada [32]. It is now believed, however, that indirect transmission of *M. bovis*, through sharing of contaminated feed or substrates, was the more likely route of transmission compared to direct contact of cattle with infected wildlife. Several studies on interactions by mammals with cattle found wildlife passing through cattle use areas or interacting directly with feed, stored feed, or water troughs with little to no direct contact between cattle and wildlife. Continued exclusion of wildlife from cattle through various changes in farms practices should be the focus of WMRAP and the exclusionary measures performed on a farm are dependent upon the host species in a respective country or region experiencing TB in cattle. 

General farm practices have also been responsible for recurring cases of *M. bovis* infection in cattle on farms in Ireland such as no mineral lick supplements being supplied to cattle (i.e., nutritional effects), purchase of a bull (i.e., cattle movements among farms), presence of cubicle housing, rough grazing areas (i.e., poor quality soil), and presence of European badgers on the farm [63]. Various farms practices can contribute to transmission of *M. bovis* through direct or indirect means (i.e., purchasing infected cattle from an outside source, use of silage clamps) that are often difficult to confirm definitively as being responsible for transmission of *M. bovis*. Cattle housed indoors rather than in pastures should be avoided to prevent within-herd transmission especially between cattle brought into a farm from an outside source to decrease chance of direct contact of infected cattle or deposition and subsequent survival of *M. bovis* in indoor facilities [65, 66]. 

The type of cattle operations (i.e., dairy, beef) posed different threats to transmission of *M. bovis* because the practices differ based on the product desired for the different operations. Mixed dairy and beef operations typically move cattle between operations more so than single dairy or beef operations to maintain adequate animals to meet production standards [76]. The increased risk of TB associated with movement of cattle onto farms from markets or farm sales seems logical and an outside source of TB infection has been documented for farms that have cattle positive for *M. bovis* in Canada, UK, Ireland, and US [63, 64, 77, 84]. 

Additional practices not included in WRMAP include landscape configuration and surrounding habitat types and additional wildlife activity on farms that have had cattle test positive for *M. bovis*. Mathews et al. [85] found a decreased risk of *M. bovis* in cattle herds on farms that practiced “nature friendly management” such as the presence of ungrazed wildlife strips and greater availability, width, and continuity of hedgerows. Although wildlife strips and hedgerows may be more important to disease transmission in European badgers, Kaneene et al. [13] documented a decrease in risk of *M. bovis* in cattle with an increase in the amount of open range on case-control farms in Michigan. Although the WRMAP addresses the proximity of livestock to “good deer habitat,” additional details on habitat types and configuration may be necessary.

Surveillance of opossum and raccoon on farms that have had cattle test positive for *M. bovis* 2 years after depopulation of cattle found mammals, other than white-tailed deer, that were infected with *M. bovis* (D. Marks, Wildlife Services, unpublished data). Although shedding of *M. bovis* by opossum was not investigated, persistence of *M. bovis* on depopulated farms from environmental sources or survival of *M. bovis* in opossum for several years suggested that constant exposure to *M. bovis* was occurring. Research findings that rely on inoculation of a single dose [37, 38] or where animals were inoculated with multiple doses [39], may not mimic the proper infective dose or duration of exposure to *M. bovis* that is occurring in the natural environment. Where free-ranging mammals may be experiencing continuous or repeated exposures to infection over weeks or months should be a focus of future research. The conflicting results of research on medium-sized mammals, low sample sizes, inability of laboratory studies to mimic exposure to *M. bovis* in free-ranging animals, lack of research on shedding by free-ranging animals, and badgers as reservoirs in New Zealand and Europe suggest that more research on medium-sized mammals as reservoirs is warranted in North America. 

## 4. Summary

The WRMAP created by the MDARD for use on farms in the upper lower peninsula of Michigan was designed following years of interviews, surveys, research, and anecdotal observations of deer on cattle farms in North America [10, 33, 72]. Combined with knowledge of badger use of farms in the UK and Ireland and brushtail possum in New Zealand, decreasing disease transmission through changed farm practices was a logical first step in eradicating *M. bovis* in Michigan. Since the hunter harvested deer was found with *M. bovis* in 1994, the ban on supplemental feeding of deer and reductions of deer herds has considerably reduced *M. bovis* prevalence in deer and on-farm risk mitigation programs have reduced the risk of farm breakdowns. 

Regardless of the efforts of management agencies in Michigan, several cattle farms experience an *M. bovis* breakdown each year within and outside of Michigan's TB core area. Additional practices to prevent access by medium-sized mammals to stored feed and cattle feed troughs could be included in WRMAP. Although the ability of mammals other than deer to shed *M. bovis* in nasal and salivary secretions and excrement is not well documented in Michigan, medium-sized mammalian species are primary reservoirs of *M. bovis* in New Zealand and Europe. Reoccurrence of *M. bovis* on farms depopulated of cattle would suggest an environmental or mammalian host source of reinfection as several farms have become reinfected with *M. bovis* on ≥2 separate occasions often spanning 3–7 years between re-infection. Assessing the potential for reservoirs other than white-tailed deer is necessary due to the current low prevalence of *M. bovis* in white-tailed deer. The low prevalence has resulted from the reduction in the number of deer, changing management priorities, and the very few direct and indirect interactions between white-tailed deer and cattle on farms in Michigan ([31, 86] National Wildlife Research Center, unpublished data). An understanding of the use of farms by Virginia opossum has been initiated but additional research on medium-sized mammals is needed in Michigan [87, 88]. 

 Research has demonstrated considerable shedding of *M. bovis* by terminally ill free-ranging badgers and brushtail possum, but there have not been any similar observations in comparable-sized free-ranging mammals in Michigan. Surveillance for *M. bovis* infection in mesocarnivores in Michigan has found that numerous species may be spillover hosts (Table 2) but research on shedding by infected free-ranging animals, other than white-tailed deer, has not been reported (but see [83]). Several wild animal species are known to use stored feed and farms buildings (e.g., barns, feed silos; [31, 87, 89]) so the potential for deposition of contaminated excreta is possible. Until additional research is conducted on potential reservoirs of *M. bovis* other than deer and more information is known on environmental persistence of *M. bovis* (e.g., in silage or grass clamps) in Michigan, the current WRMAP has provided sound guidance for on-farm mitigation of disease transmission. Requesting the assistance of farmers to protect stored feed, undertake appropriate feeding protocols, decrease cattle use of preferred deer habitat, and the banning of supplemental feeding of wild deer would the most appropriate initial steps to decrease *M. bovis* transmission should future outbreaks occur in previously TB-free states or provinces. 

From the literature review, we have identified additional measures that can be added to the WRMAP. The data to justify their implementation may not be currently available to assess our suggestions, predominately the transport of cattle from farm to farm. Although strict regulations are in compliance for the most part, farmers would need to be completely forthcoming with that information in order to assess whether or not export or import of cattle within Michigan's TB core area is actually contributing to continued presence of the disease. The reduction in density and prevalence of *M. bovis* in deer, the primary wildlife reservoir in Michigan, and breakdowns on previously unaffected farms along with recurrent breakdown on previously infected farms would suggest that other sources of infection are still involved in transmission of *M. bovis*. The very low risk of breakdown (i.e., 1 farm per year) continues to make determining the source or sources of infection very problematic.

## Figures and Tables

**Table 1:** Shedding of *Mycobacterium bovis* in experimentally and naturally infected free-ranging wildlife species.

Species	Study type	Shed	Author
Crow *Corvus brachyrhynchos*, starling *Sturnus vulgaris *	Laboratory inoculation	Negative fecal samples, concluded no shedding occurs	Butler et al. [34]

Pigeon *Columba livia *	Laboratory inoculation	Positive fecal samples for at least 60 days after inoculation	Fitzgerald et al. [36]

Mallard ducks *Anas platyrhynchos *	Laboratory inoculation	Negative fecal samples, concluded no shedding occurs	Fitzgerald et al. [35]

Brushtail possum *Trichosurus vulpecula *	Laboratory inoculation of wild caught individuals	Transmission between infected brushtail possum and controls was noted with gross lesion distribution consistent with aerosol transmission	Corner et al. [16]

Virginia opossum *Didelphis virginianus *	Laboratory oral inoculation	1 Virginia opossum fecal culture tested positive after 1 day after inoculation and another on day 31 after inoculation	Diegel et al. [37]

Virginia opossum *Didelphis virginianus *	Laboratory aerosol inoculation of wild caught individuals	Only 1 of 12 Virginia opossum had a positive fecal sample during the 90 study	Fitzgerald et al. [38]

Vole *Microtus pennsylvanicus*, mice *Mus musculus*, rat *Rattus norvegicus *	Laboratory inoculation	Positive samples of fecal cultures from the Meadow vole only	Clarke et al. [40]

European badger *Meles meles *	Samples from free-ranging groups	Positive samples from urine, feces, and bite wounds	Chambers et al. [42]

European badger *Meles meles *	Samples from free-ranging animals	Positive samples from sputum, urine, feces, and bite wounds	Clifton-Hadley et al. [2]

European badger *Meles meles *	Necropsies of animals found dead	Concluded that *M. bovis* could be shed via sputum, urine, and feces	Clifton-Hadley (cited in Gallagher et al. [90]

European badger *Meles meles *	Laboratory inoculation	Positive samples from urine and found in feces for 165–1305 days	Little et al. [41]

Feral ferret *Mustela furo *	Examination of captured free-ranging individuals	Oral cavity was the most common site of excretion of *M. bovis*; further positive samples of shedding came from tracheobronchial, urine, feces, and mammary gland samples	Lugton et al. [43]

Raccoon *Procyon lotor *	Inoculated individuals with single oral doses of *M. bovis* (ranging from 30 to 1.7 × 10^5^ cfu, five daily oral doses (ranging from 10 to 1 × 10^5^ cfu), or a single intravenous dose of 1 × 10^5^ cfu	Low doses of *M. bovis* in saliva or nasal secretions but no shedding in urine or feces	Palmer et al. [39]

Coyote *Canis latrans *	Samples from free-ranging individuals that tested tissue positive for *M. bovis *	Authors concluded that shedding was minimal based on negative culture samples from oral, nasal, and feces samples	Berentsen et al. [45]

Red deer *Cervus elaphus *	Samples from free-ranging individuals	Low shedding in feces and in nasal, pharyngeal, and tracheal swabs but no shedding in urine	Lugton et al. [46]

Marsh deer *Blastocerus dichotomus *	Esophageal-pharyngeal fluids from 53 free-ranging individuals	Concluded no shedding through this route	Luna et al. 2003 [47]

Domestic cattle *Bos taurus *	Inoculation (low dose, 10^4^ cfu; high dose 10^6^ cfu; intranasal and intratracheal inoculation) of calves in a laboratory setting	Nasal shedding was detected in 21 of 24 animals, but no calves given a low-dose shed	McCorry et al. [50]

White-tailed deer *Odocoileus virginianus *	Laboratory inoculation of mature female white-tailed deer with intratonsilar instillation of 2 × 10^3^ (low dose) or 2 × 10^5^ (high dose) cfu of *M. bovis. *	Authors conclude that the results indicated that *M. bovis* persists in tonsilar crypts for prolonged periods and can be shed in saliva and nasal secretions	Palmer et al. [48]

White-tailed deer *Odocoileus virginianus *	Laboratory inoculation and co-mingling	Positive at 63 (nasal swab), 90 (oral), and 113 (rectal) days post inoculation; positive at 69 (oral and nasal) days post co-mingling with inoculated; positive sample in feed on day 63 and 150 post inoculation, positive sample on hay on day 90 and 210 days post inoculation	Palmer et al. [49]

**Table 2:** Surveillance studies identifying all species collected and analyzed that had cultures that were tested for *Mycobacterium bovis* in North America.

Common name Scientific name	Positive samples/total samples		
	Witmer et al. [89]	O'Brien et al. [9]	Bruning-Fann et al. [5]
Study dates	2002–2004	1996–2003	1996–1999
Black bear *Ursus americanus *		7/214	1/42
Badger *Taxidea taxus *	0/4	0/46	0/2
Beaver *Castor canadensis *	0/61	NA	NA
Bobcat *Felis rufus *	0/3	4/57	0/8
Coyote *Canis latrans *	0/2	18/375	6/106
Deer mouse *Peromyscus maniculatus *	0/24	NA	NA
Domestic cat *Felis catus *	0/10	0/35	NA
Domestic dog *Canis canis *	NA	0/1	NA
Domestic rabbit *Oryctolagus cuniculus *	0/1	NA	NA
Eastern chipmunk *Tamias striatus *	0/66	NA	NA
Eastern cottontail *Sylvilagus floridanus *	0/41	NA	NA
E. gray squirrel *Sciurus carolinensis *	0/26	NA	NA
Eastern mole *Scalopus aquaticus *	0/1	NA	NA
Fox squirrel *Sciurus niger *	0/17	NA	NA
Gray fox *Urocyon cinereoargenteus *	1/1	0/5	0/1
House mouse *Mus musculus *	0/62	NA	NA
Long-tailed weasel *Mustela frenata *	NA	0/1	NA
Meadow jumping mouse *Zapus hudsonius *	0/7	NA	NA
Meadow vole *Microtus pennsylvanicus *	0/77	NA	NA
Mink *Mustela vison *	NA	0/5	NA
Muskrat *Ondatra zibethicus *	0/5	NA	NA
N. flying squirrel *Glaucomys sabrinus *	0/1	NA	NA
Porcupine *Erethizon dorsatum *	0/71	0/1	NA
Raccoon *Procyon lotor *	5/203	8/333	2/48
Red fox *Vulpes vulpes *	NA	3/29	1/5
Red squirrel *Tamias striatus *	0/58	NA	NA
Red-backed vole *Clethrionomys gapperi *	0/3	NA	NA
River otter *Lutra canadensis *	NA	0/10	NA
Snowshoe hare *Lepus americanus *	0/23	0/1	NA
S. bog lemming *Synaptomys cooperi *	0/1	NA	NA
S. flying squirrel *Glaucomys volans *	0/3	NA	NA
Striped skunk *Mephitis mephitis *	0/46	0/21	NA
13-lined ground squirrel *Spermophilus tridecemlineatus *	0/4	NA	NA
Virginia opossum *Didelphis virginianus *	4/135	2/379	0/54
White-footed mouse *Peromyscus leucopus *	0/66	NA	NA
White-tailed deer *Odocoileus virginianus *	0/2	NA	NA
Woodchuck *Marmota monax *	0/10	NA	NA
Woodland vole *Microtus pinetorum *	0/3	NA	NA
